# Supplementary material for: Cerebellar-driven cortical dynamics can enable task acquisition, switching and consolidation
Source: Nat Commun. 2024 Dec 30;15:10913. doi: 10.1038/s41467-024-55315-6 (PMC11686095; doi:10.1038/s41467-024-55315-6)
Supplement: Supplementary file 1 — Supplementary Information [file 41467_2024_55315_MOESM1_ESM.pdf]

Supplementary Information

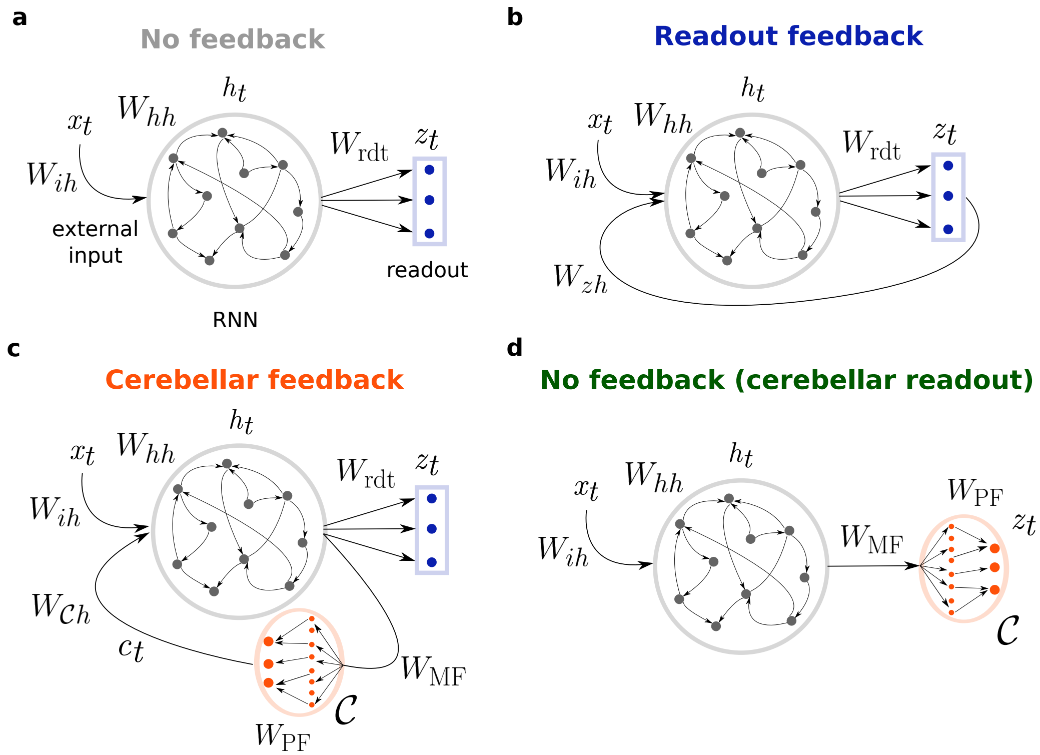

Figure S1. Different model architectures (extension of Fig. 1). **a** *No feedback*; temporal input is fed to a cortical RNN (grey) and a linear readout layer (blue) produces the final model output. **b** *Readout feedback*; now there is a feedback loop in which the RNN also receives readout predictions as extra input[1,2]. **c** *Cerebellar feedback*; a copy of RNN activity is sent to a distinct but connected cerebellar network $\mathcal{C}$, which then returns its predictions back to the RNN as extra input. **d** *No feedback with cerebellar readout*; like in c a cerebellar network is attached to the RNN, but now it is used directly as the final readout and there is no "cortico-cerebellar loop". Model activity and weight vectors are represented with the same notation as Eqs. 3 and 4 (see also Table 1).

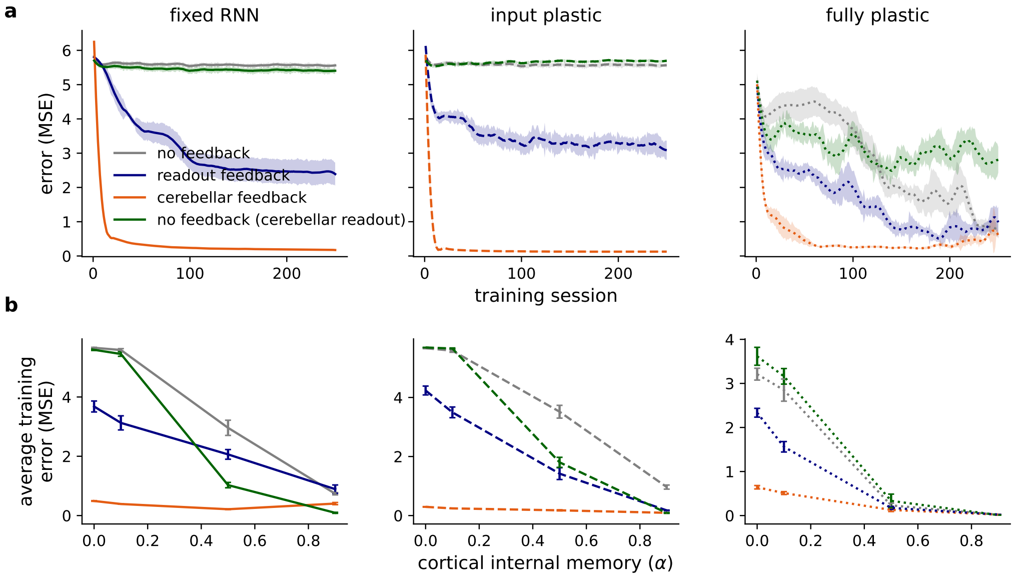

Figure S2. Model learning in the line drawing task. a Training curves (cortical internal memory $\alpha = 0.1$) for the different models with fixed (left), input plastic (middle) and fully plastic (right) RNN. Green denotes the model where no feedback is applied to the RNN but the readout network (usually linear) now has the same architecture as the cerebellar network. **b** Average error over training across different cortical internal memory α.

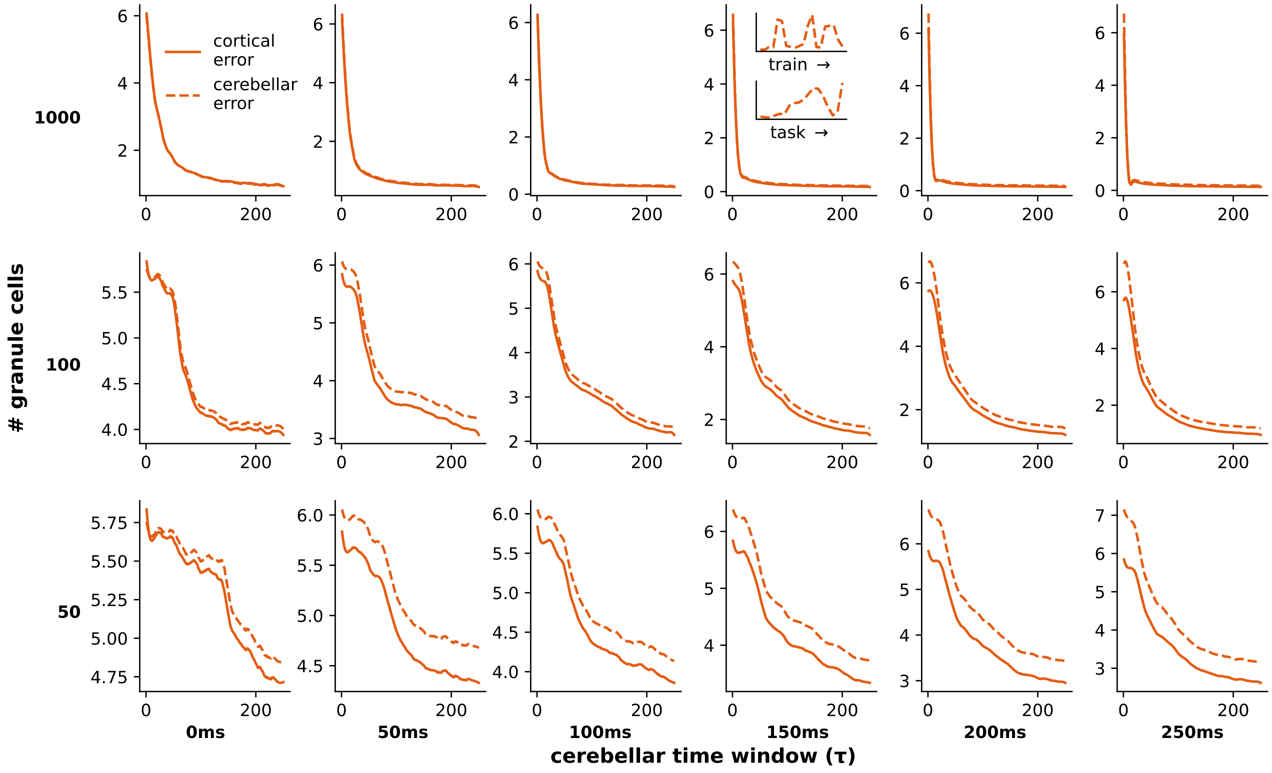

Figure S3. Training curves over different cerebellar parameters. Learning curves for the cortical network (solid line) and cerebellar network (dotted line) for the line drawing task. On each miniplot the x-axis represents the training session and y-axis the mean-squared error. The cerebellar error for an example seed is shown in the inset of the model conditions used in the main text (1000 granule cells, time window $\tau = 150ms$), over different task examples during training (upper) and over time within one task example (lower).

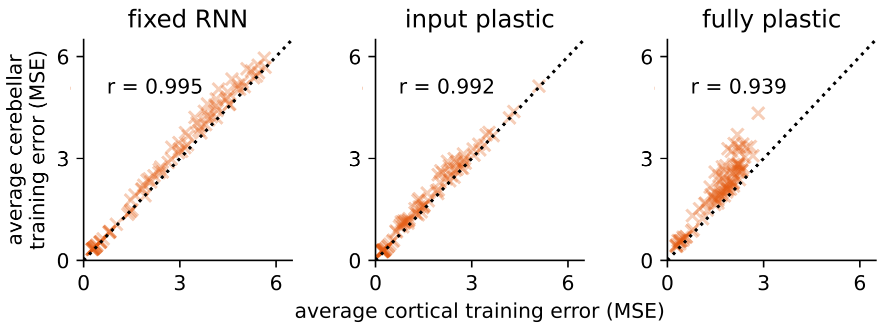

Figure S4. Integrated errors for the cortical network against the cerebellar network for the line drawing task. Each point denotes a specific cerebellar parameter configuration (1 of the 18 in Fig. S3) and initialisation seed (1 of 5). r denotes the Pearson correlation.

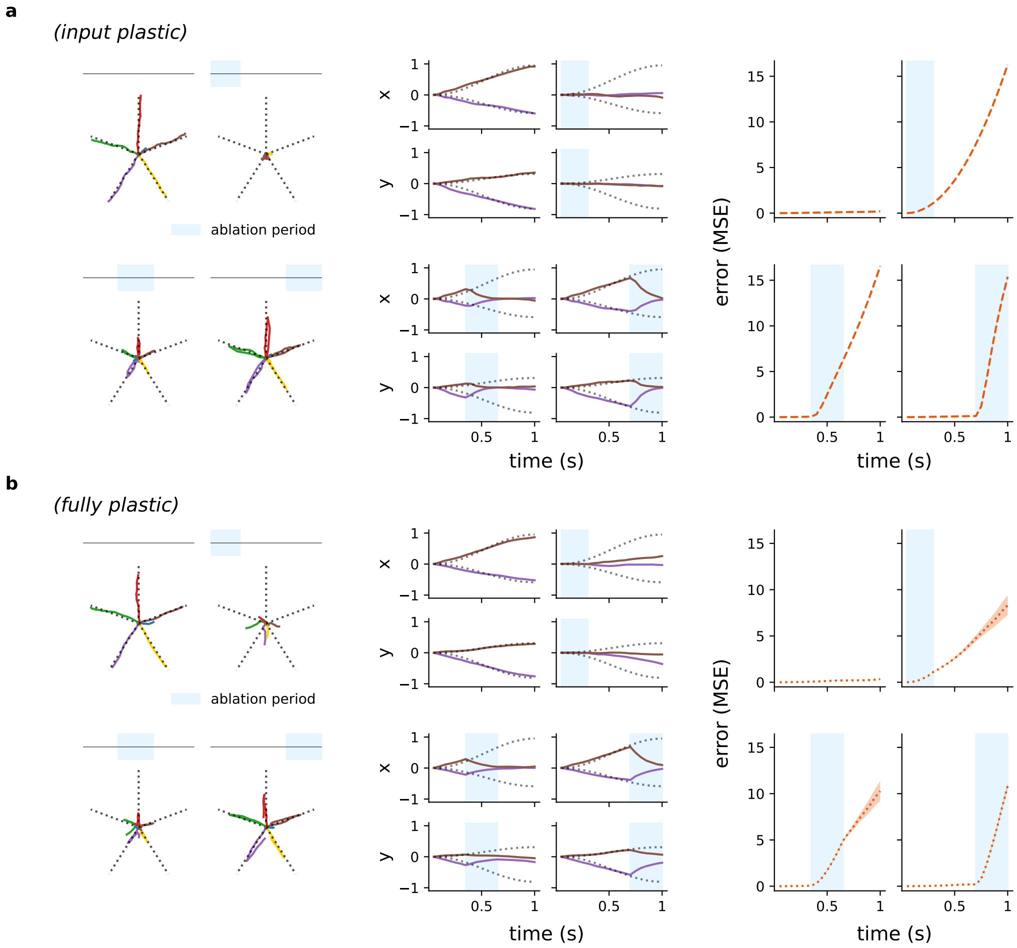

Figure S5. Ablation results for the input and fully plastic RNN. Model output (left, middle) and error (right) for example line drawing input under cerebellar ablation for an **a** input plastic and **b** fully plastic RNN. For the corresponding fixed RNN case see Fig. 2h-j.

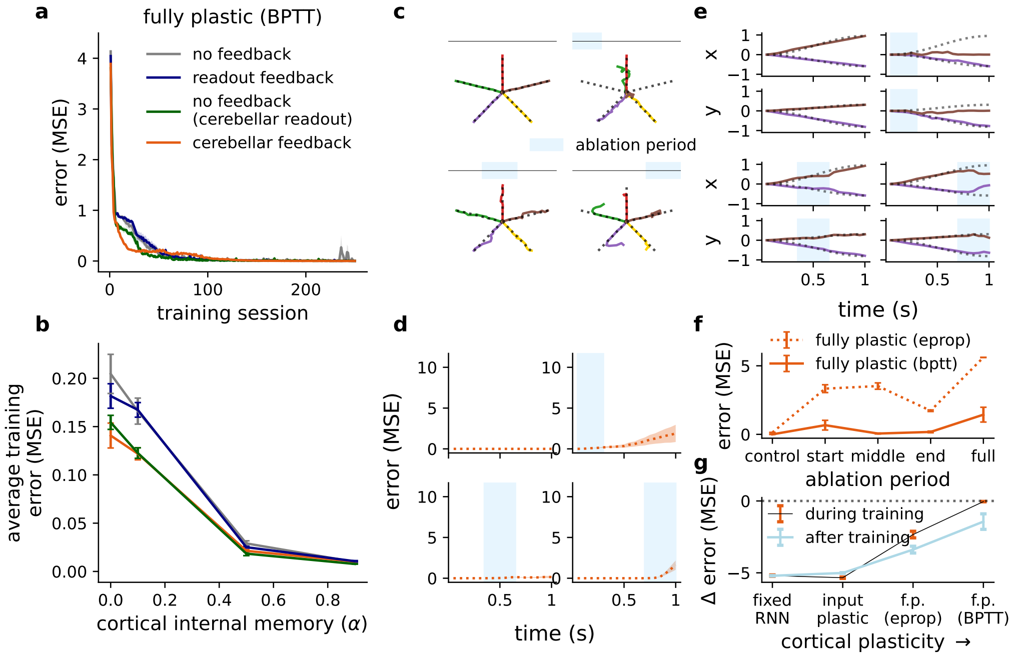

Figure S6. Model learning and dynamics when the cortical RNN is trained with backpropagation through time (BPTT) in the line drawing task (fully plastic). **a** Training curves (cortical internal memory $\alpha = 0.1$) for the different models with BPTT. Green denotes the model where no feedback is applied to the RNN but the readout network (usually linear) now has the same architecture as the cerebellar network. **b** Average error over training across different cortical internal memory α. **c-e**, Effect of cerebellar ablation at different time periods; **c** model output, **d** x, y components of model output, **e** model error. **f** Average error for different degrees of plasticity and ablation periods (left to right) as in c-e; fully plastic model trained with eligibility traces (as presented in main text) is shown for reference. **g** Change in task error for models with versus without cerebellar component during and after training.

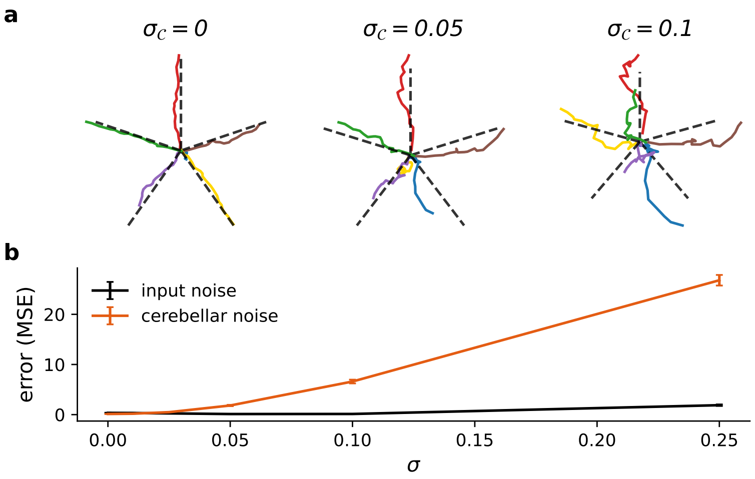

Figure S7. Cerebellar noise induces ataxic-like impairments. **a** Output for trained models on the line drawing task under different levels of cerebellar noise $\mathbf{c}_t^{\text{noise}} = \mathbf{c}_t + \xi_t$ with $\xi_t \sim \mathcal{N}(\mathbf{0}; \sigma_C^2 \mathbf{I})$; blue output is for “no go” cue (where model is trained to remain at zero). **b** Model error under various degrees of input and cerebellar noise.

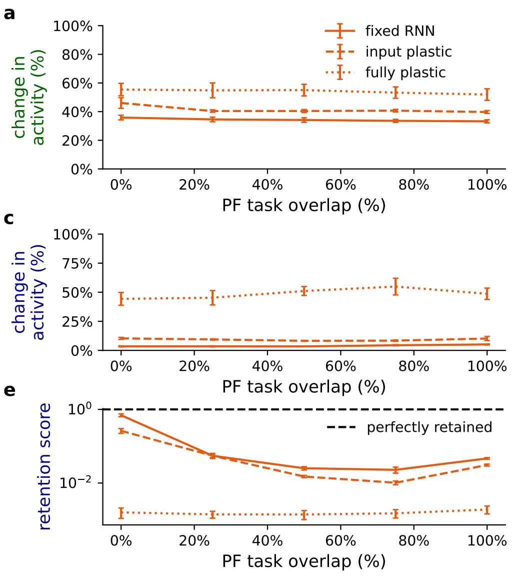

Figure S8. Multi-task learning and switching across different levels of parallel fibre (PF) overlap. (**a**,**b**) Change in (a) activity and (b) covariance in RNN population between the line-drawing task 1 (baseline) and task 2 which is a curl-field variant. (**c**, **d**) Change in (c) activity and (d) covariance in RNN population between task 1 (baseline) and task 1 (post re-learning) after switching back from task 2. **e** Task 1 retention score, which is computed as the error of task 1 during baseline over the error at the first trial after switching back to task 1.

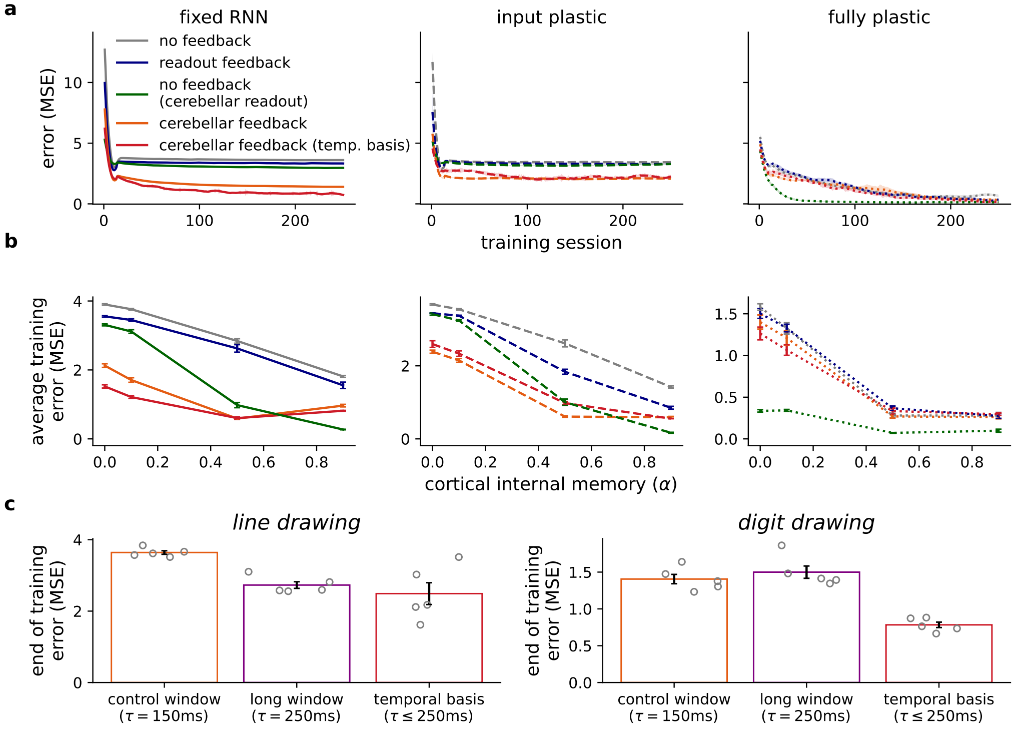

Figure S9. Model learning in the digit drawing task. a Training curves (cortical internal memory $\alpha = 0.1$) for the different models with a fixed (left), input plastic (middle) and fully plastic (right) RNN plasticity assumptions. Green denotes the model where no feedback is applied to the RNN but the readout network (usually linear) now has the same architecture as the cerebellar network. **b** Average error over training across different cortical internal memory α. **c** Model error (fixed RNN; $\alpha = 0.1$) at the end of training (averaged over last 10 training sessions) for different cerebellar time windows for (left) line drawing task (cf. Fig. 2) and (right) digit drawing task.

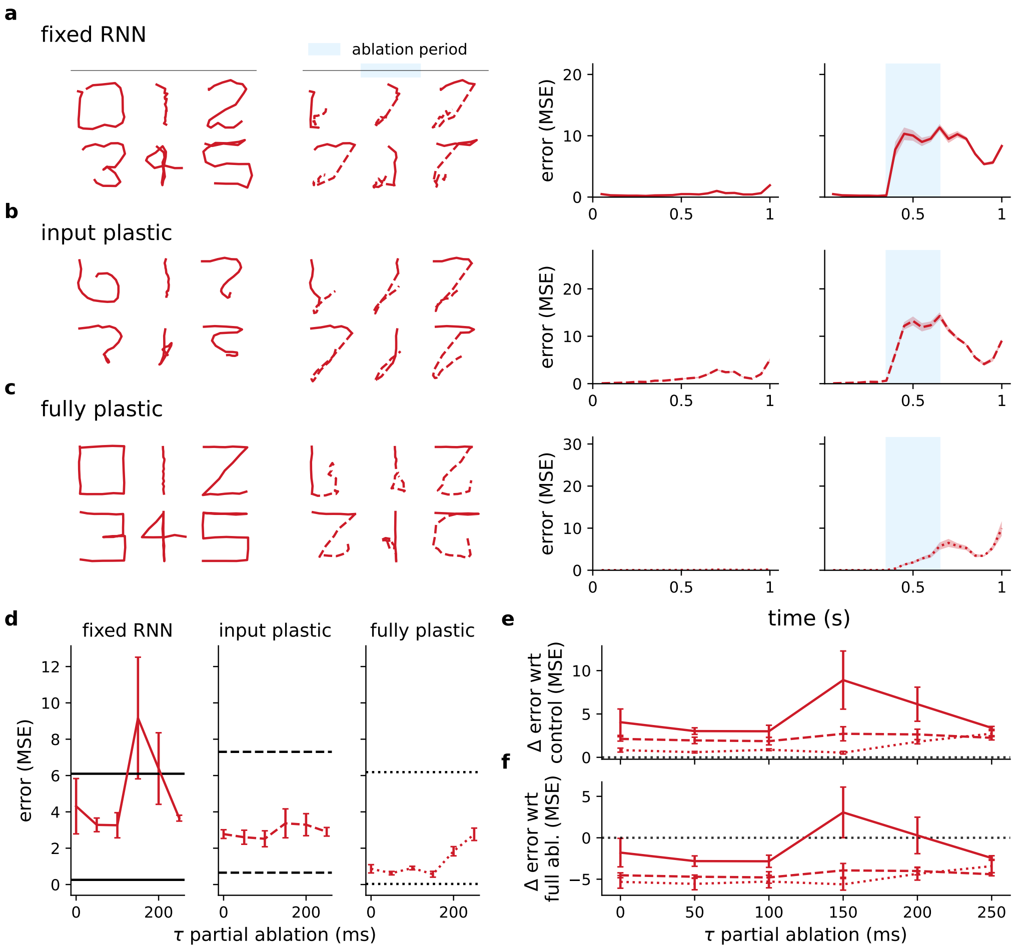

Figure S10. Ablation results for the digit drawing task. **a-c** Model output (left) and error (right) for digit drawing input under cerebellar ablation for a (a) fixed, (b) input plastic, and (c) fully plastic RNN. Model output shown after cerebellar ablation with dotted lines. **d-f**, Effect of cerebellar partial ablation, where only cerebellar predictions trained with a specific time window τ are removed. **d**, Error under partial ablation across individual τ for different RNN plasticity conditions; bottom black line denotes control performance, top black line denotes performance under full cerebellar ablation (cf. a-c). **e,f** Change in performance under partial ablation versus (e) control and (f) full ablation conditions

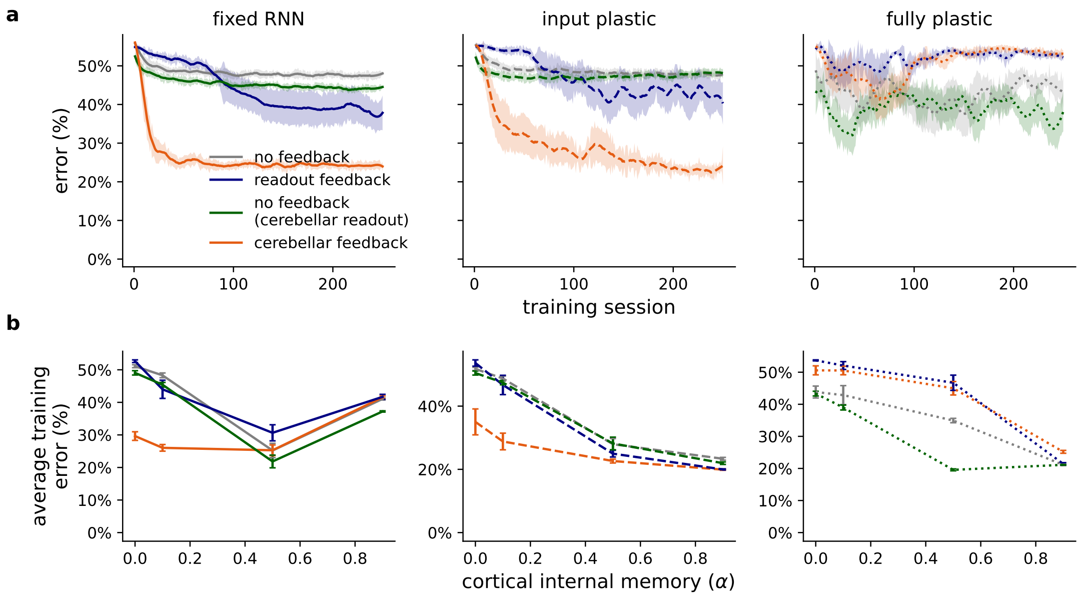

Figure S11. Model learning in the evidence accumulation task. a Training curves (cortical internal memory $\alpha = 0.1$) for the different models with a fixed RNN (left), input plastic (middle) and fully plastic (right) RNN. Green denotes the model where no feedback is applied to the RNN but the readout network (usually linear) now has the same architecture as the cerebellar network. **b** Average error over training across different levels of cortical internal memory α.

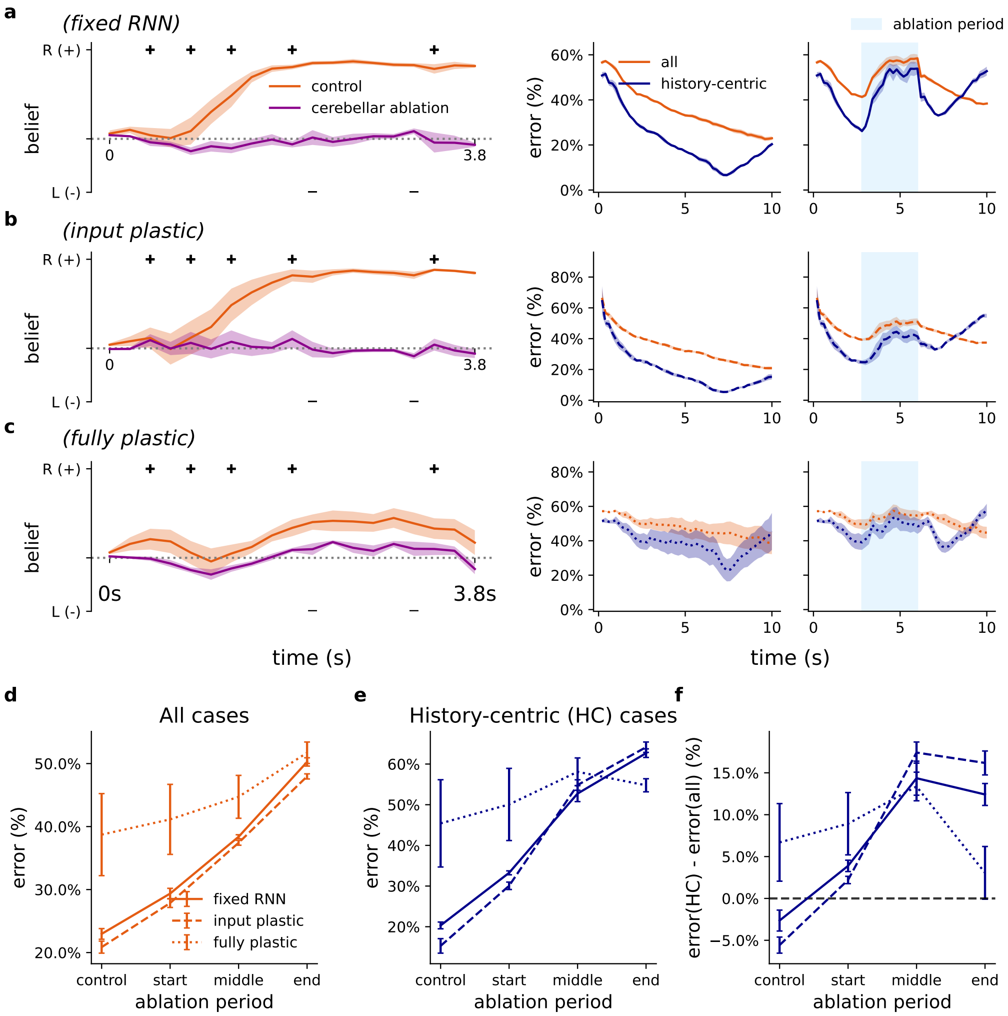

Figure S12. Additional cerebellar ablation results for evidence accumulation task. (**a**-**c**) Model output (left) and error (right) with and without cerebellar ablation (model output shows full cerebellar ablation case) for (a) fixed, (b) input plastic, and (c) fully plastic RNN. **d** The error for different ablation periods across these RNN plasticity conditions over all test examples. **e** The error for different ablation periods across different RNN plasticity conditions, but only over "history-centric" inputs. **f** The difference between d and e.

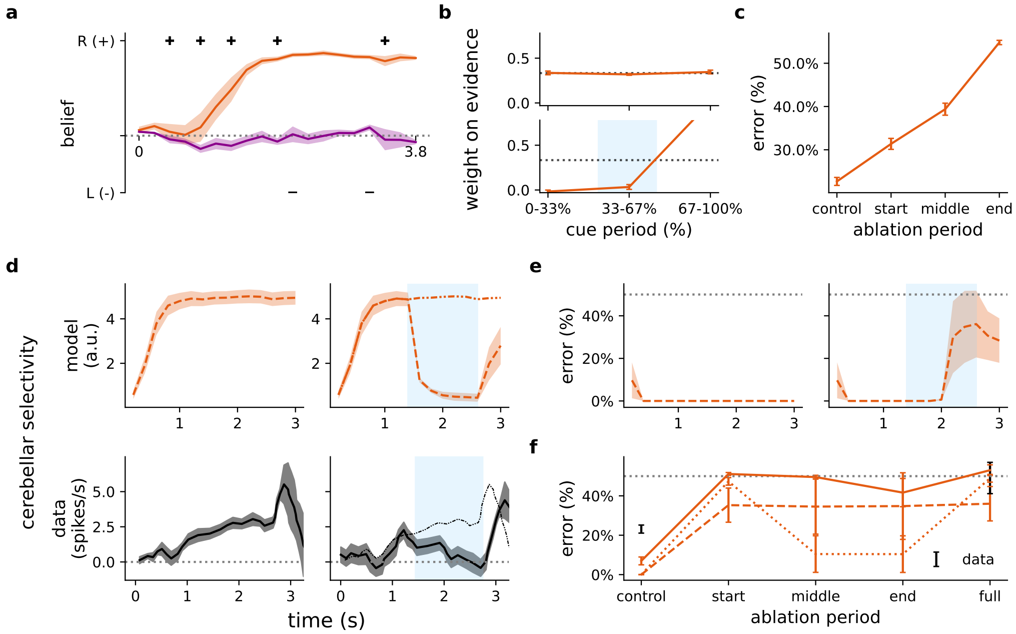

Figure S13. Effect of cortical ablation in working memory tasks. In this analysis 75% of cortical RNN neurons are silenced (after training). (**a**-**c**) Cortical ablation during the evidence accumulation task (fixed RNN). **a** Model output without (orange) and without (purple) cortical ablation over the whole task period. **b** Normalised regression weights at different periods of input presentation (cue) during control (upper) and cortical ablation (lower; ablation period denoted in blue) conditions. **c** Model error under different ablation periods. (**d**-**f**) Delayed association task (input plastic RNN). **d** Cue selectivity in the cerebellar network during the delay period without (left) and with cortical ablation (ablation period denoted in blue) conditions for example input in model (upper panels) and experimental data (lower panels) reproduced from Gao et al. [3]. **e** (Cortical) model error during delay period with (left) and without (right) cortical ablation. **f** Average error from cortical ablation at different periods during the task delay period and different degrees of plasticity. Experimental data shown in black.

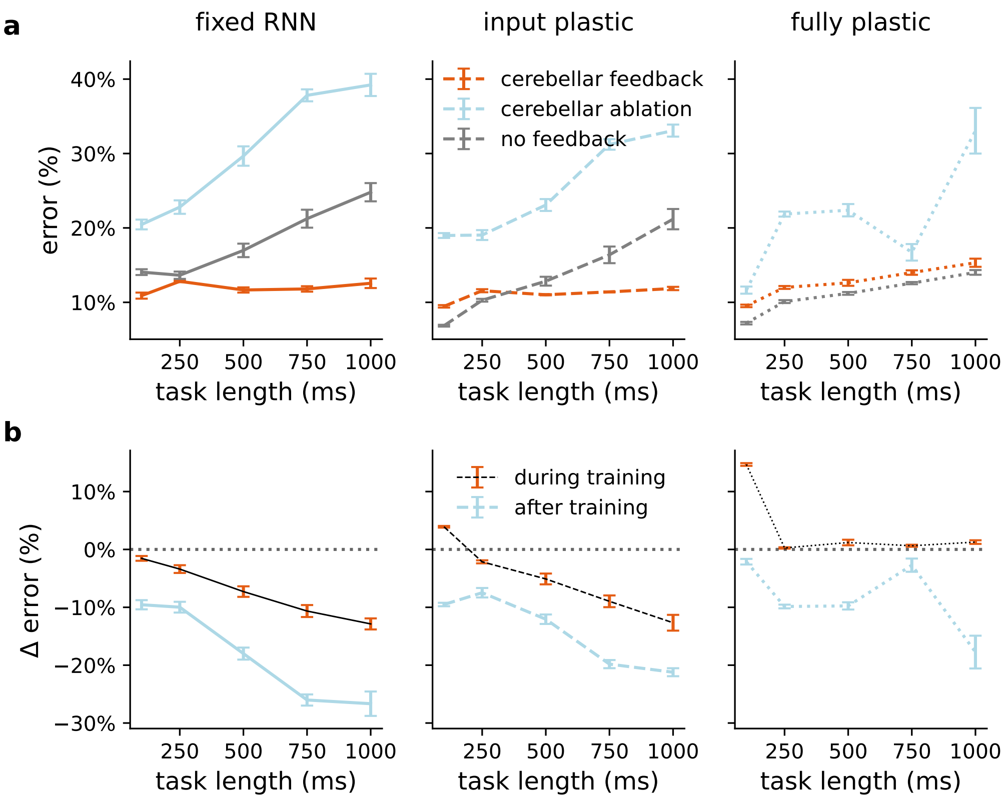

Figure S14. Effect of cerebellar ablation on evidence accumulation task with varying cue durations. **a** Test error over different cue durations for models trained with cerebellar feedback (orange), models trained with cerebellar feedback but now subject to cerebellar ablation (light blue), and models trained without cerebellar feedback (grey), with a fixed (left), input plastic (middle) or fully plastic (right) RNN. **b** Average change in training error over different cue durations for models with versus without cerebellar component during and after training.

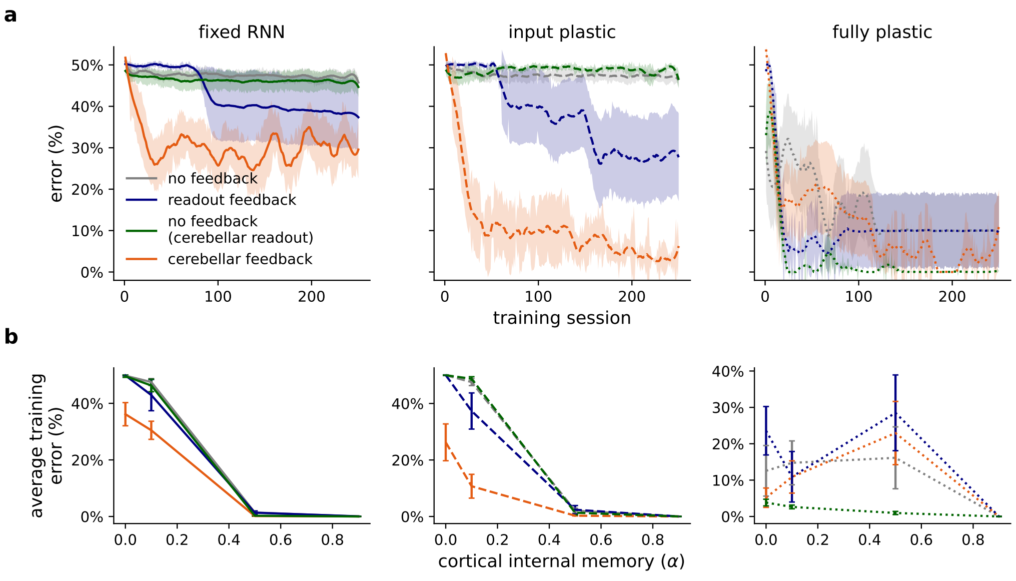

Figure S15. Learning curves in the delayed association task. a Training curves (cortical internal memory $\alpha = 0.1$) for the different models with a fixed (left), input plastic (middle) and fully plastic (right) RNN. Green denotes the model where no feedback is applied to the RNN but the readout network (usually linear) now has the same architecture as the cerebellar network. **b** Average error over training across different cortical internal memory α.

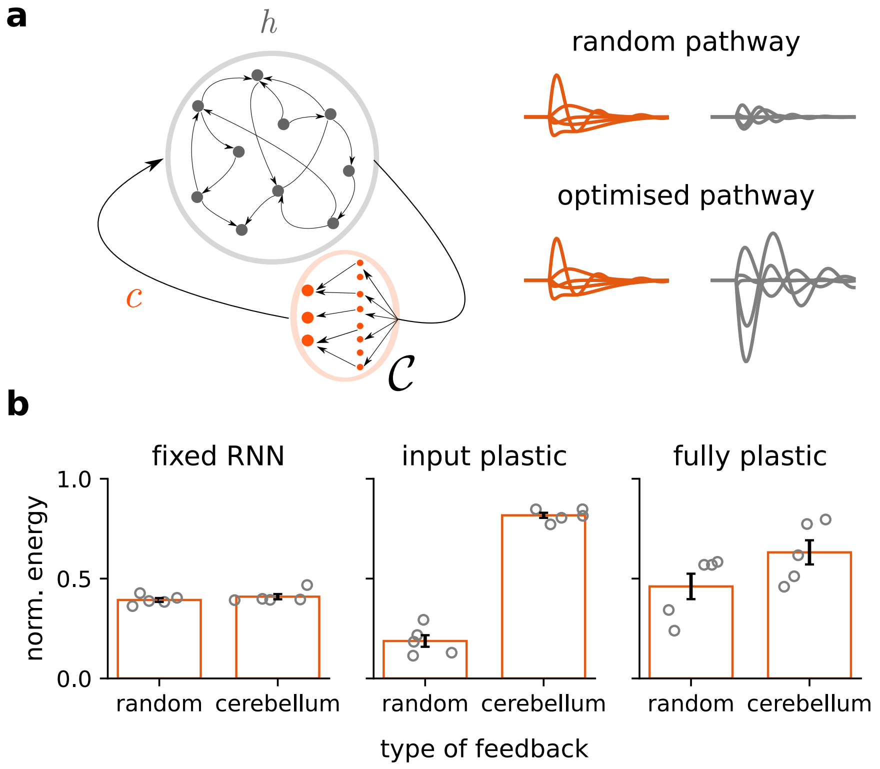

Figure S16. A control-theoretic perspective of the cortico-cerebellar loop. a Illustrative schematic of cerebellar (orange) and cortical (grey) activities. Depending on the cerebellar-cortical connectivity $\mathbf{W}_{\mathcal{C}h}$, the same cerebellar output **c** might suppress (top right) or amplify (bottom right) RNN trajectories. **b** The energy (see Methods) generated by random and cerebellar feedback for models trained with varying degrees of plasticity in the delayed association task (Fig. 6). The energy is normalised by the maximum possible energy generated by inputs that achieve the greatest cortical response (see Methods).

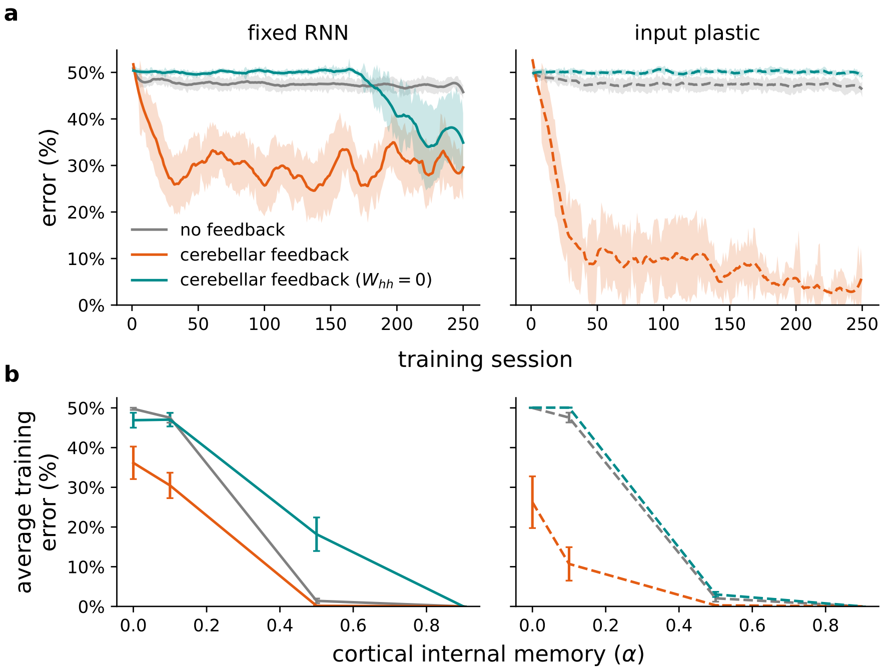

Figure S17. Cerebellar feedback with cortical recurrent connectivity is necessary to learn long-range temporal associations. **a** Training curves (cortical internal memory $\alpha = 0.1$) with cerebellar feedback but zero recurrent weights ($\mathbf{W}_{hh} = 0$) with a fixed (left) and input plastic (right) RNN. **b** Average error over training across different cortical internal memory α.

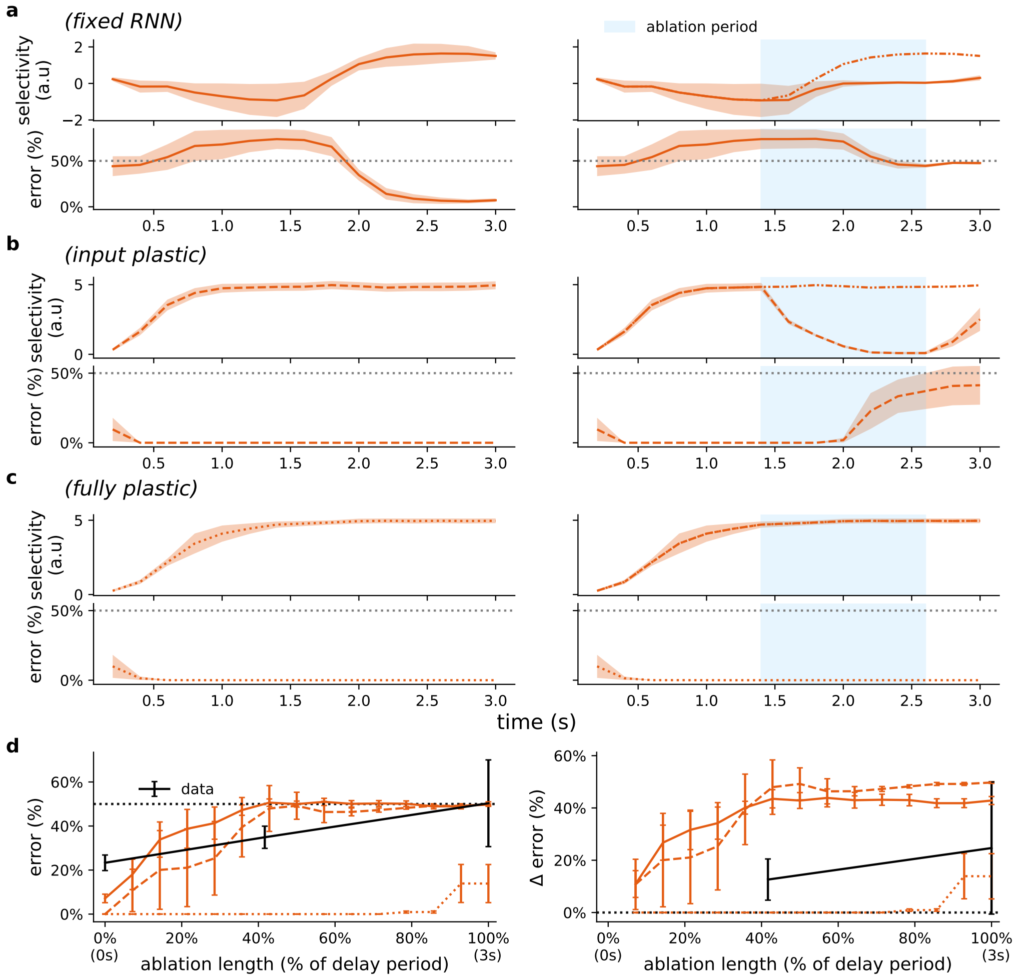

Figure S18. Additional cerebellar ablation results for the delayed association task. (**a**-**c**) Model output (top) and error (bottom) for the delayed association task without (left) and with (right) cerebellar ablation with a (**a**) fixed, (**b**) input plastic, and (**c**) fully plastic RNN. Thin line after ablation shows control model. **d** Model error as a function of ablation length (centred around the middle of the delay period). Experimental data reproduced from Gao et al. [3]. Dotted black line denotes chance.

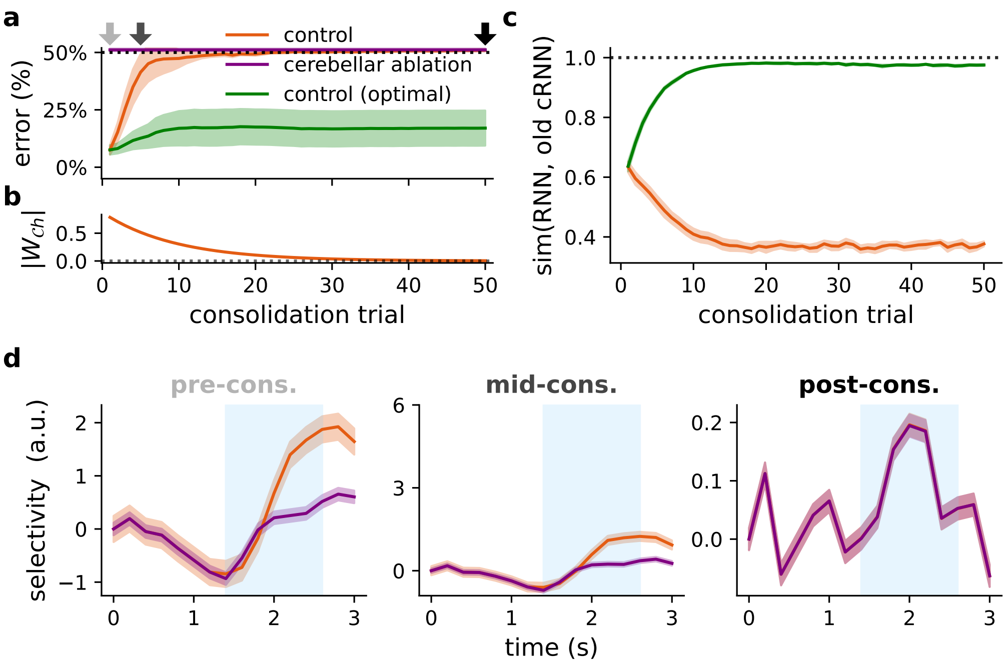

Figure S19. Cerebellar-to-cortical consolidation of the delayed association task with fixed RNN models. **a** Accuracy of control and cerebellar ablation conditions (dotted line denotes chance) and the corresponding **b** strength of the cerebellar-cortical pathway (W_{Ch}) over consolidation. Green denotes control condition with theoretically optimal learning rule. **c** Cosine similarity between cortico-cortical input and total cortical input (i.e. cerebellar-cortical and cortico-cortical inputs) pre-consolidation. Similarity of the consolidation model is shown in orange and the optimal consolidation model in green. **d** Model selectivity for example (external) input in control and cerebellar ablation conditions at different stages of the consolidation process; colour coded by arrow times in a.

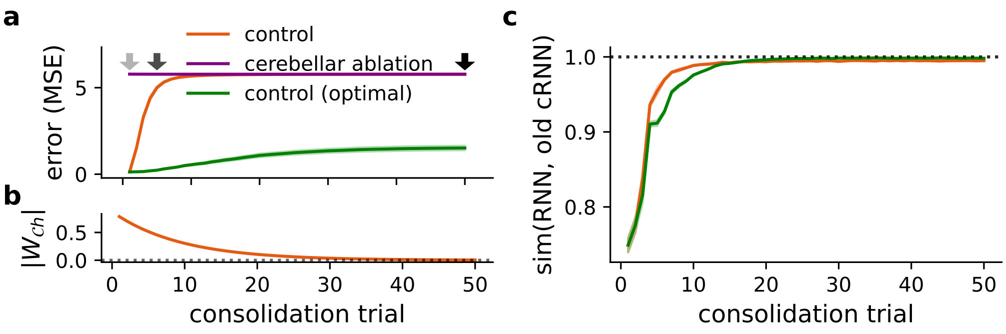

Figure S20. Cerebellar-to-cortical consolidation in linedraw task (fixed RNN). **a** Error (mean-squared error) of control and cerebellar ablation conditions and the corresponding **b** strength of the cerebellar-cortical pathway (W_{Ch}) over consolidation. Green denotes control condition with theoretically optimal learning rule. **c** Cosine similarity between cortico-cortical input and total cortical input (i.e. cerebellar-cortical and cortico-cortical inputs) pre-consolidation. Similarity of the consolidation model is shown in orange and the optimal consolidation model in green. Note that even though the similarity between these models is high, their small differences result in significant changes in the overall trajectory of cortico-cerebellar activity, resulting in poor final performance.

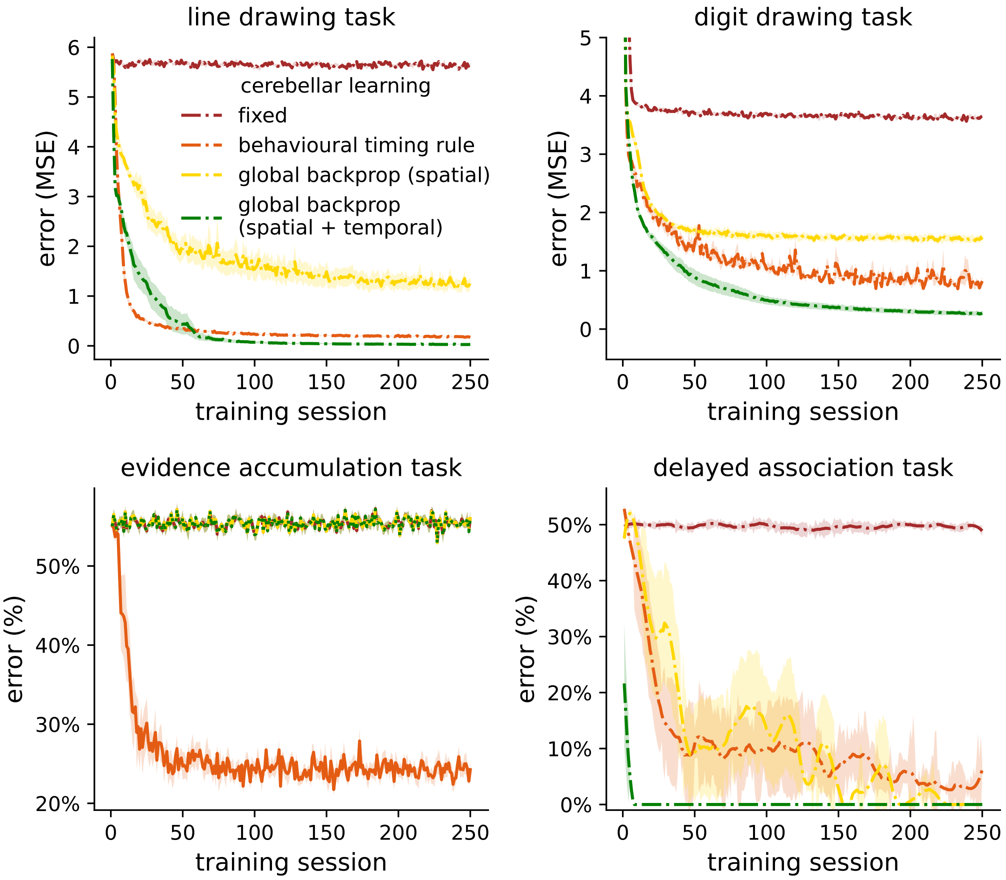

Figure S21. Performance of models with cerebellar feedback when the cerebellar network learns with backpropagated teaching signals. In this scenario the cerebellum is optimised to directly minimise the cortical error E_t via global (cortico-cerebellar) backpropagation through the cortical readout network and RNN (cf. Fig. 1c). Two forms of backpropagation are considered: 1. backpropagation through space (spatial), which only considers the effect of cerebellar feedback on the current error 2. backpropagation through space and time (spatial + temporal; i.e. BPTT), which also takes into account the effect of cerebellar feedback on future cortical errors. The timing rule that we employ in the main text is shown for comparison (behavioural timing rule), as well as the case for which there is no cerebellar learning at all (fixed). In line with the main experimental results presented in the text, in all tasks a fixed cortical RNN is considered, except for the delayed association task in which an input plastic RNN is used. Note that for the evidence accumulation task, only our behavioural timing learning rule successfully learns. It is perhaps surprising that cerebellar learning via BPTT fails in this scenario; we speculate this is due to the longer sequence length involved in this task which can create problematic conditions for the transmission of gradients[4].

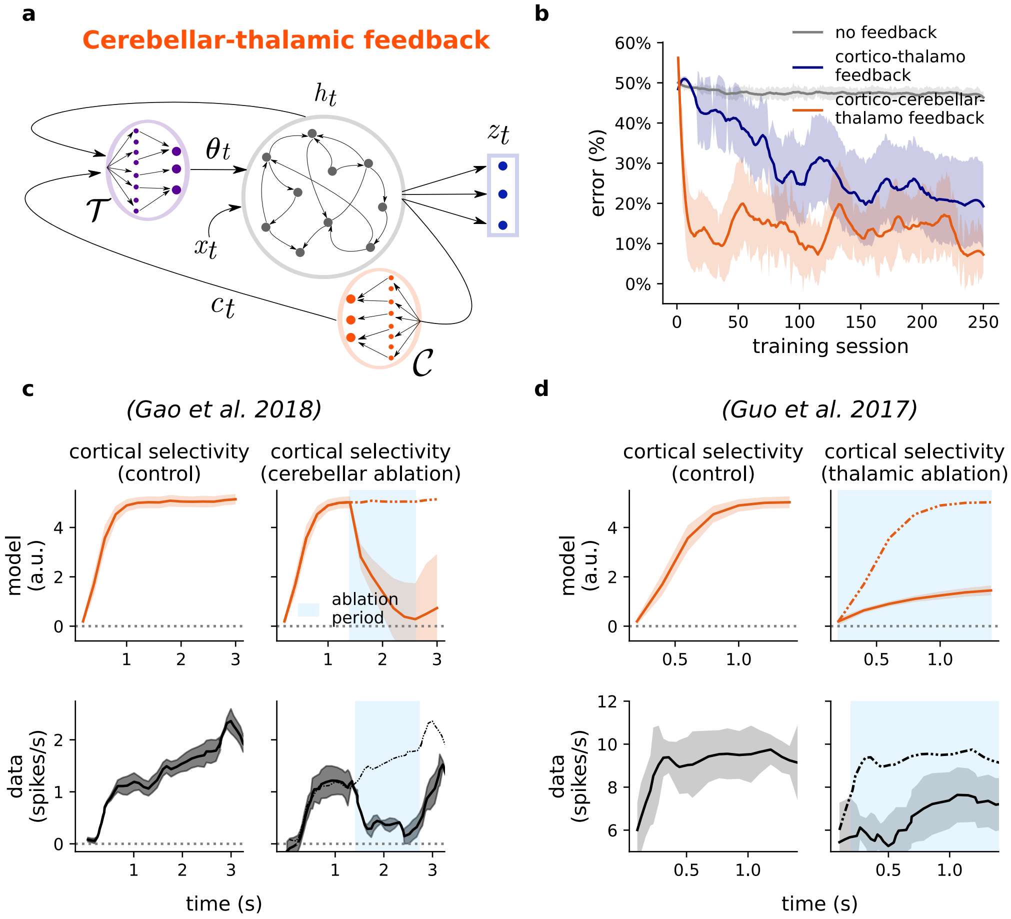

Figure S22. Cortico-cerebellar-thalamic circuit can replicate experimental results for both cerebellar and thalamic ablation. **a** Model architecture for the cortico-cerebellar-thalamic loop. The cortex projects a copy of its activity $\mathbf{h}_t$ onto both a cerebellar ($\mathcal{C}$) and thalamic ($\mathcal{T}$) network. The cerebellar prediction $\mathbf{c}_t$ is passed onto the thalamic network, which in turn provides a signal θ_t onto the cortical RNN. For implementation details see Methods. **b** Learning curves for models trained with cerebellar-cortical feedback; cortical-thalamic feedback is shown for reference, in which the cerebellar-thalamic (and therefore cerebellar-cortical) interaction is abolished. **c** Effect of cerebellar ablation, as per[3], on the cortico-cerebellar-thalamic model. **d** Effect of (partial; see Methods) thalamic ablation, as per[5], on the cortico-cerebellar-thalamic model. Data from[5] was acquired for selectivity from contra-preferring neurons (see Extended data Figure 7).

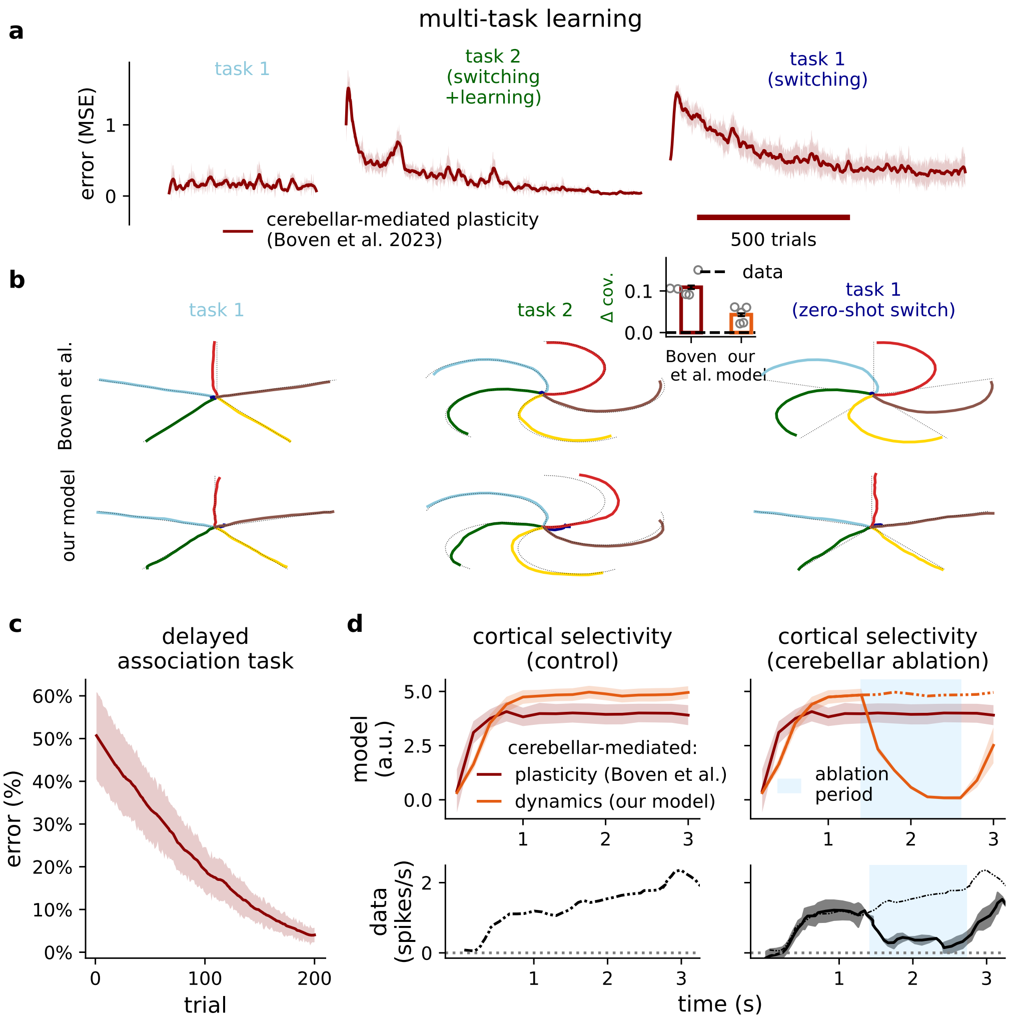

Figure S23. Comparison of our model against a model of cerebellar-mediated cortical plasticity as per Boven et al. 2023[6]. In this model the same general architecture of the cortico-cerebellar loop is used, except that now the cerebellar output is used in the learning rule of the cortical RNN (see[6] for details). **a,b** Model performance (a) and output (b) during multi-task learning (cf. Fig. 3) **c,d** Model training performance (c) and effect of cerebellar ablation after training (d) in the delayed association task. We highlight that whilst in each of these paradigms the model can successfully learn the task, the model fails to capture the fast switching or single-trial cortico-cerebellar dependency as captured by our model.

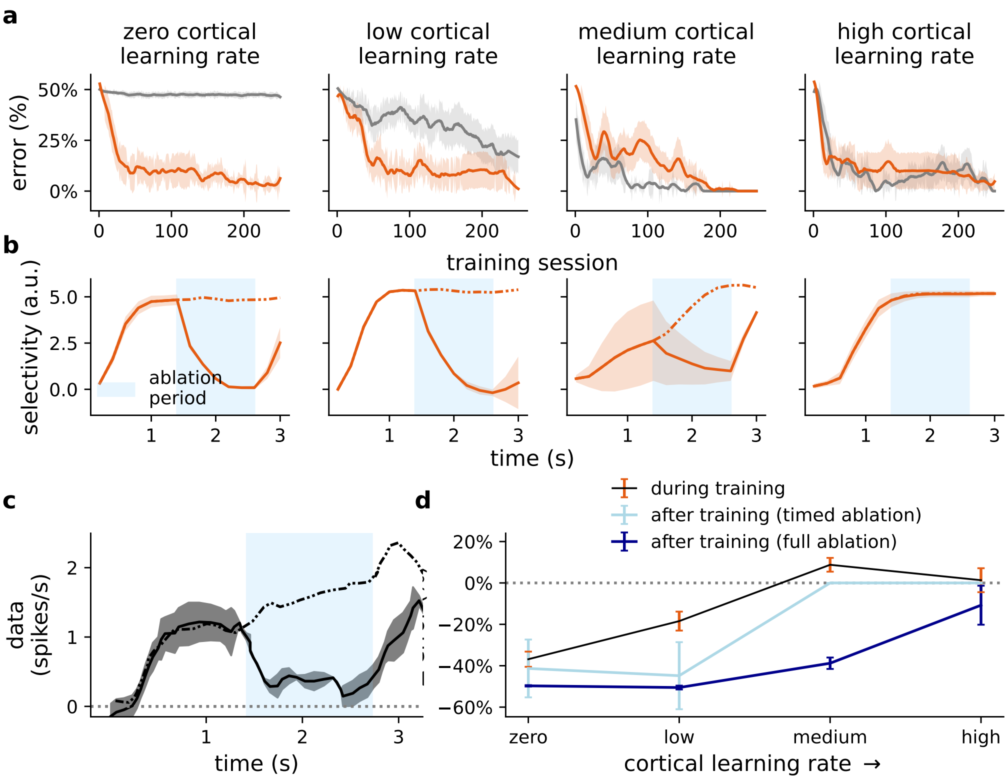

Figure S24. Impact of cortical learning rates on cortico-cerebellar interactions. To further demonstrate that the plasticity of recurrent cortical synapses determines the importance of the cerebellar network, we tested the model under a range of cortical (recurrent) learning rates. **a** Learning curves in the delayed association task for cortico-cerebellar model (orange) and cortical-alone model across different cortical learning rates. **b** Task selectivity in the model with (solid line) and without (dashed line) ablation of the cerebellar module. **c** Cortical neural data with (solid line) and without (dashed line) optogenetic inhibition of the cerebellum[3]. **d** Summary plot of change in error across different cortical learning rates.

References

[1] Herbert Jaeger and Harald Haas. Harnessing nonlinearity: Predicting chaotic systems and saving energy in wireless communication. *science*, 304(5667):78–80, 2004.

[2] David Sussillo and Larry F Abbott. Generating coherent patterns of activity from chaotic neural networks. *Neuron*, 63(4):544–557, 2009.

[3] Zhenyu Gao, Courtney Davis, Alyse M Thomas, Michael N Economo, Amada M Abrego, Karel Svoboda, Chris I De Zeeuw, and Nuo Li. A cortico-cerebellar loop for motor planning. *Nature*, 563(7729):113, 2018.

[4] Sepp Hochreiter. The vanishing gradient problem during learning recurrent neural nets and problem solutions. *International Journal of Uncertainty, Fuzziness and Knowledge-Based Systems*, 6(02):107–116, 1998.

[5] Zengcai V Guo, Hidehiko K Inagaki, Kayvon Daie, Shaul Druckmann, Charles R Gerfen, and Karel Svoboda. Maintenance of persistent activity in a frontal thalamocortical loop. *Nature*, 545(7653):181–186, 2017.

[6] Ellen Boven, Joseph Pemberton, Paul Chadderton, Richard Apps, and Rui Ponte Costa. Cerebro-cerebellar networks facilitate learning through feedback decoupling. *Nature Communications*, 14(1):1–18, 2023.
