# Supplementary material for: Cerebellar-driven cortical dynamics can enable task acquisition, switching and consolidation
Source: Nat Commun. 2024 Dec 30;15:10913. doi: 10.1038/s41467-024-55315-6 (PMC11686095; doi:10.1038/s41467-024-55315-6)
Supplement: Supplementary file 2 — Peer Review file [file 41467_2024_55315_MOESM2_ESM.pdf]

nature portfolio

Peer Review File

Cerebellar-driven cortical dynamics can enable task acquisition, switching and consolidation

Corresponding Author: Dr Rui Ponte Costa

This manuscript has been previously reviewed at another journal. This document only contains information relating to versions considered at Nature Communications.

Version 0:

Reviewer comments:

Reviewer #1

(Remarks to the Author)
The authors have satisfactorily addressed most of my technical questions. Some conceptual disagreements remain; for instance, the assumption that the cortex is a suboptimal learning region still seems like a strawman to me. However, I would recommend the publication of this study as it is, as the paper has already undergone thorough reviews and the modeling part is solid. As a purely modeling study, it serves its purpose of advocating the importance of the cerebellum in supporting cerebral learning. How exactly cerebellum contributes to cortical learning should be further studied by other experimental labs in the future.

(Remarks on code availability)

Reviewer #2

(Remarks to the Author)
I appreciate the changes the authors have made to the manuscript. I think the claims are now much more reasonable, and the paper presents a hypothesis that may lead to experimental predictions.

(Remarks on code availability)
The code is well-documented.

Open Access This Peer Review File is licensed under a Creative Commons Attribution 4.0 International License, which permits use, sharing, adaptation, distribution and reproduction in any medium or format, as long as you give appropriate credit to the original author(s) and the source, provide a link to the Creative Commons license, and indicate if changes were made.
In cases where reviewers are anonymous, credit should be given to 'Anonymous Referee' and the source.
The images or other third party material in this Peer Review File are included in the article's Creative Commons license, unless indicated otherwise in a credit line to the material. If material is not included in the article's Creative Commons license and your intended use is not permitted by statutory regulation or exceeds the permitted use, you will need to obtain permission directly from the copyright holder.
To view a copy of this license, visit https://creativecommons.org/licenses/by/4.0/
